# Toward Unity and Inclusion in the Clinical Workplace: An Evaluation of Healthcare Workforce Belonging During the COVID-19 Pandemic

**DOI:** 10.7759/cureus.29454

**Published:** 2022-09-22

**Authors:** David Gordon, Kathryn Achuck, Danielle Kempner, Rebecca Jaffe, Dimitrios Papanagnou

**Affiliations:** 1 Sidney Kimmel Medical College, Thomas Jefferson University, Philadelphia, USA; 2 Jefferson College of Rehabilitation Sciences, Thomas Jefferson University, Philadelphia, USA; 3 Internal Medicine, Thomas Jefferson University, Philadelphia, USA; 4 Emergency Medicine, Thomas Jefferson University, Philadelphia, USA

**Keywords:** wellbeing, multidisciplinary teams, employee engagement, professional burnout, belonging, staff wellbeing, physician wellbeing

## Abstract

Introduction: In a challenging time for the healthcare workforce responding to the coronavirus disease 2019 (COVID-19) pandemic, it is critical to identify factors contributing to team members' feelings of “belonging” in the workplace. The Institute for Healthcare Improvement’s Quintuple Aim’s principle of improving healthcare worker well-being could be applied to explore the implications of the increased turnover and stress, which connect to components of belonging. This study applies a qualitative approach to the organizational issues impacting healthcare teams, particularly during a complex and uncertain time.

Methods: To elucidate factors contributing to belonging, we conducted a series of semi-structured interviews with an interdisciplinary cross-sectional sample of healthcare workers. Interviews were conducted with 23 total staff members in two clinical settings, the emergency department and hospital medicine groups at a large urban teaching hospital, to evaluate team members’ perspectives of the work environment.

Results: Participants discuss their degree of inclusion, excitement, challenges, and respective needs from the organization. Perspectives of workers representing varied professional roles of the healthcare team were gathered to provide robust and unique insights into initiatives that can enhance belonging in the clinical workplace.

Conclusion: Our findings provide a preliminary framework to identify strategies that can potentially reinforce collective team member belonging and consequently improve staff well-being, morale, and retention.

## Introduction

The coronavirus disease 2019 (COVID-19) pandemic has posed challenges for healthcare providers working in the clinical environment. Occupational concerns about transmitting or succumbing to COVID-19, witnessing, first-hand, the devastating effects the virus has on patients and staff, and adapting to ever-changing protocols have debilitated the psychological and mental health of the clinical workforce [1,2]. Non-occupational stressors have only compounded the burnout and impaired team dynamics. Compassion fatigue remains rampant, impacting team cohesion, staff retention, and the overall burden on staff. Consequently, we have witnessed record-high staff turnover across clinical disciplines [3]. Today’s healthcare delivery relies on interdisciplinary teams, in which proper organizational dynamics and team development can help to establish the team’s success [4].

In light of these challenges, the Institute for Healthcare Improvement has made the case for healthcare organizations to uphold the Quintuple Aim of Healthcare. The Quintuple Aim is a five-part mission to improve population health, enhance the patient experience, reduce healthcare costs, improve care team well-being, and advance health equity [5]. In this context, through the utilization of semi-structured interviews, we revisit the question of what it means to “belong” in a healthcare team - on a personal, professional, and institutional level.

## Materials and methods

To evaluate healthcare belonging, we conducted workplace-based interviews with clinical teams working in inpatient and outpatient clinical settings at an academic hospital in an urban setting. Through a literature search of prior studies investigating belonging, a series of variables contributing to belonging were identified [6-9]. These variables were extracted and arranged thematically to synthesize aspects of belonging relating to ethnocultural and identity factors, workplace and economic factors, and mental wellness factors (Table 1).

**Table 1:** Summary aspects contributing to belonging

Aspect of belonging	Possible enhancements	Possible barriers
Ethnocultural and identity-based [10-12]	Inclusion of personal identity in team culture, team reinforces values, community organizations	Language, discrimination, xenophobia, microaggressions
Workplace and economic-based [8,11,13]	Full-time work, home ownership, adjustments to pay model and rate, group harmony, and respect	Organizational issues, cost of living, obligations outside work, differing professional foci
Mental wellness-based [13,14]	The personal value derived from team involvement and mission, support for personal needs, conscience, openness to team	Communication issues, increased burdens during and outside work, stress, burnout

Regarding one’s personal identity, it is important to consider individual identification with the team culture, one’s alignment with their personal cultural values, and one’s feeling of integration within the organization [10-12]. If one encounters discrimination, xenophobia, or microaggressions, the sense of ethnocultural and identity-based belonging declines. Within the workplace, hours worked, homeownership, pay, and group mutual respect influence the economic component of belonging [8,11,13]. Organizational issues, including obligations outside of work and divergence in professional goals, also decrease this component of belonging. Finally, the personal value derived from involvement in the team’s mission, sense of personal support, and willingness to communicate with the team contribute to the psychosocial wellness aspect of belonging [13,14]. When teams encounter communication issues, increased stress, or role strain, it worsens wellness within the workplace. Across each theme, aspects of inclusion were tantamount to a sense of belonging [6-9].

For each aspect of belonging, prior literature explored factors contributing to strain in the sense of belonging. Open-ended questions were adapted from the literature to gauge workplace satisfaction as it relates to each of the aforementioned components of belonging (Table 2).

**Table 2:** Healthcare belonging questionnaire

#	Question
1	What is your hospital department? What is your role on the department's team?
2	How long have you been in this role?
3	What makes you feel valued at work?
4	Inclusion can be defined as “cherishing the whole identity of a person”. How do you feel the workplace supports your inclusion?
5	What, if anything, prevents you from feeling fully included?
6	What makes you excited to work here?
7	Are there things that make you feel not excited to work here? (If yes, what?)
8	To what extent has the COVID-19 pandemic changed how you feel at work?
9	What would be the best thing that your organization could do to help meet your needs?
10	What is the best thing others on your team could do to help meet your needs?

Using the belonging interview questionnaire, we completed a series of semi-structured interviews at a large urban teaching hospital in Philadelphia, Pennsylvania. These interviews were conducted with a convenience sample of interdisciplinary personnel across two care settings in April 2022. The research team approached staff (N = 23) in the emergency department (n_ED_ = 10) and hospital medicine groups (n_HM_ = 13). Both groups represent highly interdisciplinary teams, where staff frequently collaborate with other team members from different health professions [4]. A cross-section of the staff, including staff from many professional disciplines, was sampled.

Eligibility was based on having staff or student status within the healthcare team. The team member may be patient- and/or non-patient-facing. Students rotating from professional programs were included in the interview. Interviews were conducted using the questionnaire as a guide, with the research team asking follow-up questions to probe deeper into responses. Each interview was approximately five to 15 minutes and took place during a staff member’s typical hospital shift. No incentive was provided for staff to participate. As this research was limited to information-gathering interviews without personal information, this study was considered exempt from human subjects review.

## Results

**Figure 1:**
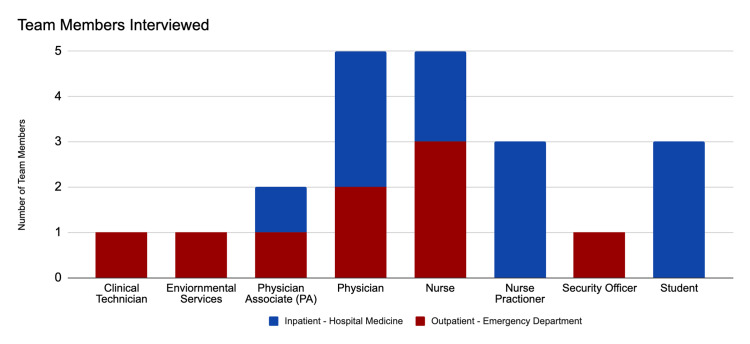
Professional roles of team members interviewed

Through these interviews, we gained new insights into staff challenges, perspectives, and identities to help elucidate features contributing to workplace belonging as staff respond to the COVID-19 pandemic. A total of 23 team members completed the semi-structured interviews. The interviews were conducted with a broad range of personnel, representing the many disciplines of the healthcare professional team within each department (Figure 1). We assessed eight unique roles in the healthcare team including a nurse, nurse practitioner, environmental service staff, security, technician, physician associate, physician, and student. Professional identity, by itself, may represent a strong contributor to perspectives on belonging as team members’ roles shape the structure of the interdisciplinary team.

**Figure 2:**
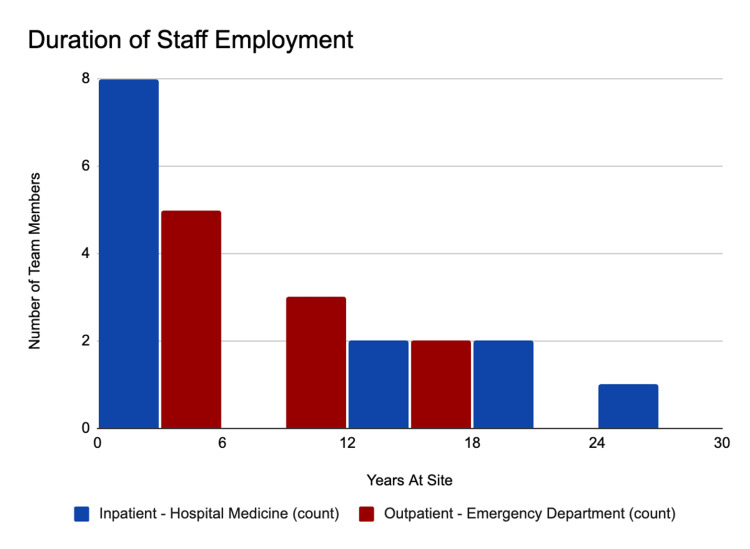
Duration of staff employment

Of those sampled, the average duration of employment was 6.7 years for the emergency department group and 8.3 years for the hospital medicine group (Figure 2). The median duration of staff employment was 5.5 years for the emergency department group and 9.25 years for the hospital medicine group. In general, physicians had the longest duration of staff employment. All students interviewed started clinical rotations during the pandemic.

Our random selection of staff yielded employees largely new to the organization. When students were included, the median employment duration of the hospital medicine group interviewed decreased to two years. In this sample, 43% of the staff interviewed joined the team after the pandemic was already underway. This is consistent with national data identifying increased staff turnover during the pandemic, as well as increased staff leaving the healthcare workforce [15].

Interview responses were summarized based on values, inclusion, motivators, organizational contributions, and team member contributions (Table 3). Across professions, staff highlighted workplace inclusion, acknowledgment, respect, and collegiality as positively influencing their experiences in the organization.

**Table 3:** Summary of staff responses

Belonging theme	Outpatient team - emergency department	Inpatient team - hospital medicine
Values	Patient care; acknowledgment of challenges; incentives; learning from others on the team; positive reinforcement	Patient care; coordination; formal and informal feedback channels; feeling of having a voice; kindness and respect
Enhancements to inclusion	Treat how others want to be treated; include all team members; elevate different backgrounds and identities; relative seniority; backup from others; contribute to organizational change; accommodation of schedule requests; group text message	Solicitation of input; value learners; schedule flexibility; everyone contributes; respect; voice heard amongst my supervisors; social events
Challenges to inclusion	High team turnover; tendency to form cliques; communication gaps; emotional detachment to work; admin and clinical disconnect; siloed work	Support differs between night and day; lack of institutional knowledge; workplace disputes; micromanagement; lack of meeting invites; admin and clinical disconnect; colleagues interested in personal bonds
Motivators	Camaraderie and co-workers; compensation; daily variety in work; shared team humor; newly designed workplaces; autonomy; improvement in COVID-19 cases	People on the team; co-workers; autonomy; respect; workplace-based learning; collegiality; nature of the job
Organizational contributions	Retaining those with experience; teaching; reconciling competition with travel nurses; increased compensation; improved security through facility design; increase communication outside meetings; improved feedback and teaching; acknowledgment and respect; clear communication	Incentives and benefits package; clear communication; feedback; improved scheduling and time off; increased staffing; increased compensation; workload balancing; additional resources provided
Team member contributions	Team cooperation and mutual respect; transparent communication; understanding colleagues; support with kindness; seeking input from staff; advocating for inclusion; improved scheduling	Mutual respect; opportunities for mutual feedback; acknowledging learners; social events outside work; team cooperation and communication

In both clinical environments, stressors or challenges cited included the pace of work, communication barriers, and obligations outside patient care. With the emergency department group, unique stressors cited included accepted admissions boarding, the stress associated with the start of a shift, and workplace security. With the hospital medicine group, unique stressors included new teams, patients with complex social needs, and negative team member interactions.

Relating to the COVID-19 pandemic, team members reported increased stress, new perspectives, and challenges to belonging. Early on, staff cited respect for the healthcare team in a “terrifying” time. However, the climate has since changed to return to baseline levels of respect, but with additional COVID-19-related stressors. One team member noted the pandemic caused the worst “mental and physical anxiety” of their career, “changing their perspective on life.” The pandemic brought challenges in adapting, enforcing hospital policies, and changes to morale. Some cited a decline in passion and perceived compensation affecting their mindset to come to work, leading to changes in empathy and possible team strain. Nurses noted that this turnover brought new nurses with different perspectives, but also increased difficulties in tending to a patient’s bedside needs.

## Discussion

In both settings, staff values included providing the best possible care to help patients reach health goals, kindness, respect, and openness to seek opinions from others. The patient care message was noted by team members of multiple professions, ranging from physicians to environmental services. Students emphasized feeling included within the team, kindness, and respect for status as learners. For nurses and nurse practitioners, communication within the team, especially with physicians, was important. It was also important that communication resulted in feedback, either informally through co-workers reinforcing a job well done or formally through a performance evaluation structure. Overall, responses on values represented hybrid personal values, such as feedback or incentives; team values, such as communication; and organizational mission-based values, such as patient care. With additional strain from caring for others during the pandemic, the feeling that a staff member's values are being fulfilled may influence decisions on whether to remain with an organization or in the workforce.

While these responses were specific to our institution, the belonging trends along three categories (ethnocultural and identity, workplace and economic, and mental wellness) build a generalizable framework for any institution [10,11,13].

Future efforts should conduct additional samples to assess these findings specific to other organizations. Our semi-structured interview questions were intended to take less than 10 minutes so as not to interfere with clinical duties. Future work should include an in-depth analysis highlighting the effects on other aspects of personal health and wellness, such as sleep, caregiving roles, anxiety, and depression, to paint a better picture of how other factors may contribute to and be affected by healthcare belonging.

As organizations propose strategies targeted toward staff belonging, morale, and retention, it is important to share these findings with stakeholders. Stakeholders may include the entire team, which consists of a richness of backgrounds and professional perspectives. The built environment of the workplace, ranging from types of workstations to the layout of the space, may also influence perceptions of belonging. Without physical interactions with the team, staff working remotely may experience unique challenges to belonging. Research may further investigate factors affecting belonging between those who work predominantly in person and those who work virtually.

Since the beginning of the COVID-19 pandemic, multiple studies have examined the effects on the healthcare industry. A study conducted by the University of Minnesota suggests that physicians are leaving the industry at rates higher than before the pandemic and many are choosing not to return to the workforce [16]. Healthcare worker shortages are of great concern to the community and organizations. With a lack of personnel, hospitals may be unable to staff beds or the care team may take on an amplified workload, possibly leading to more errors. Additionally, staffing shortages may increase financial pressures or change organizational culture. Healthcare belonging is becoming subsequently harder to obtain, as compounding occupational and non-occupational stressors related to COVID-19 abound.

COVID-19 at its core is a global and population health issue. Given the Institute for Healthcare Improvement’s Quintuple Aim’s goal of improving population health, we must ensure that we are meeting the framework of all five tenets, which also include the healthcare personnel's well-being, health equity, the patient care experience, and per capita healthcare cost.

## Conclusions

It is only through the collaboration and teamwork of the healthcare providers that we can protect against challenging times and raise awareness of the urgency for an interdisciplinary approach to create a mutually understood, cultural definition of belonging. Through our interviews, we found that every member of the patient care team, regardless of their interprofessional role, was needed to uphold the values of the quadruple aim to maintain workplace belonging. We reviewed factors that personally motivated individual employees. Amongst the healthcare workers we talked to, many had self-selected an environment and culture that matched their dedication to caring for patients. This apparent dedication to improving the patient experience satisfies one component of the quintuple aim. To further build on the other components, i.e., reducing healthcare costs, improving team well-being, and advancing health equity, we must turn to a professional and institutional level view of the situation.

On a professional level, we identified that strained personal and team belonging prompted challenges in retention and decreased morale. However, many team members were able to set actionable tasks that we hope to use as the launching point for organizational strategies to bring about change on an institutional level, thereby improving team well-being and decreasing healthcare costs through the maintenance of experienced staff members. For any new initiative, the intervention should include policy creation centered on the quintuple aim. In an age of heightened staff turnover, a focus on personal, team, and organizational belonging may help to improve staff belonging, morale, and retention.
